# SEAHIR: A Specialized Compartmental Model for COVID-19

**DOI:** 10.3390/ijerph18052667

**Published:** 2021-03-06

**Authors:** Alexandros Leontitsis, Abiola Senok, Alawi Alsheikh-Ali, Younus Al Nasser, Tom Loney, Aamena Alshamsi

**Affiliations:** 1 Smart Dubai Department, Dubai Design District, Building 1A, Dubai P.O. Box 555995, United Arab Emirates; alexandros.leontitsis@smartdubai.ae (A.L.); younus.alnasser@smartdubai.ae (Y.A.N.); 2 College of Medicine, Mohammed Bin Rashid University of Medicine and Health Sciences, Building 14, Dubai Healthcare City, Dubai P.O. Box 505055, United Arab Emirates; abiola.senok@mbru.ac.ae (A.S.); alawi.alsheikhali@mbru.ac.ae (A.A.-A.)

**Keywords:** compartmental models, COVID-19, Erlang distribution, predictive modelling, SARS-CoV-2, SEIR

## Abstract

The SEIR (Susceptible-Exposed-Infected-Removed) model is widely used in epidemiology to mathematically model the spread of infectious diseases with incubation periods. However, the SEIR model prototype is generic and not able to capture the unique nature of a novel viral pandemic such as SARS-CoV-2. We have developed and tested a specialized version of the SEIR model, called SEAHIR (Susceptible-Exposed-Asymptomatic-Hospitalized-Isolated-Removed) model. This proposed model is able to capture the unique dynamics of the COVID-19 outbreak including further dividing the Infected compartment into: (1) “Asymptomatic”, (2) “Isolated” and (3) “Hospitalized” to delineate the transmission specifics of each compartment and forecast healthcare requirements. The model also takes into consideration the impact of non-pharmaceutical interventions such as physical distancing and different testing strategies on the number of confirmed cases. We used a publicly available dataset from the United Arab Emirates (UAE) as a case study to optimize the main parameters of the model and benchmarked it against the historical number of cases. The SEAHIR model was used by decision-makers in Dubai’s COVID-19 Command and Control Center to make timely decisions on developing testing strategies, increasing healthcare capacity, and implementing interventions to contain the spread of the virus. The novel six-compartment SEAHIR model could be utilized by decision-makers and researchers in other countries for current or future pandemics.

## 1. Introduction

In December 2019, cases of a new respiratory illness were reported from Wuhan, Hubei Province, China. This newly emerged infectious disease now named as coronavirus disease 2019 (COVID-19) is caused by the novel severe acute respiratory syndrome coronavirus-2 (SARS-CoV-2) [1,2]. The World Health Organization initially declared the COVID-19 outbreak as a Public Health Emergency of International Concern but with the spread across and within countries leading to a rapid increase in the number of cases, the outbreak was declared a pandemic on 11 March 2020 [3]. Since the turn of the millennium, there have been two major global outbreaks caused by coronaviruses; namely, SARS-CoV, 2002–2004 and Middle Eastern Respiratory Syndrome, MERS 2012–present [4,5,6]. However, the ongoing COVID-19 pandemic is the most devastating in terms of numbers of infections, morbidity, and mortality, and is a harsh reminder of the challenges posed by emerging infectious diseases [7]. The number of confirmed COVID-19 cases globally has exceeded 114 million to date and the impact of the pandemic on healthcare, social, and economic structures across the world has been unprecedented [2,7]. Indeed, strict measures to ensure physical distancing including closure of schools and businesses as well as restrictions on travel and gatherings were implemented to varying degrees in most countries [2].

Mathematical modelling is used to make predictions on the number of cases, disease spread, and duration of an epidemic, and to estimate the impact of intervention measures during infectious disease outbreaks. Although the use of mathematical models has a long history dating back to the 18th century, compartmental models were only introduced in the early 20th century [8]. The “Susceptible-Exposed-Infected-Removed” model (SEIR hereafter) is a widely used compartmental, deterministic model, developed to simplify the mathematical modelling of infectious diseases. According to SEIR, the population is assumed to be fixed and over time moving from one compartment to another with certain rates. In SEIR, the people in the Exposed compartment, are not supposed to be infectious; however, in the case of COVID-19, the literature provides evidence that even people in the Exposed compartment can be infectious [9]. Moreover, the Infected compartment is supposed to transmit the infection to the Susceptible compartment. In the case of COVID-19 this assumption partly holds. The diagnosed carriers may immediately be admitted to a hospital or sent to home isolation for approximately 14 days. On the other hand, there are non-diagnosed carriers who show no symptoms (e.g., fever, cough) and these asymptomatic cases can transmit the virus as they are not under any movement, travel, or social restrictions if they are not tested. In this paper we report a new six-compartment model that addresses the above issues and includes other factors that are specific to COVID-19, in order to have an accurate model for decision making and pandemic management.

The rest of the paper is organized as follows: In Section 2 we discuss the core assumptions of the proposed model and the modifications which address the public health interventions, and then we proceed to the formal definition as a set of ordinary differential equations. In Section 3, we discuss the details of the optimization of the free parameters of the proposed model, and the potential cases of the undetected patients. Finally, in Section 4, Section 5 and Section 6, we discuss our findings, future work, and conclusions. All the data in this paper are publicly available data from Dong et al. [10] and are also reflected in the UAE Federal Competitiveness and Statistics Authority [11] or are based on assumptions.

## 2. The SEAHIR Model Assumptions

We made the following core modelling assumptions to develop our model:

- Six compartments: Susceptible-Exposed-Asymptomatic-Hospitalized-Isolated-Removed (SEAHIR).
- The Susceptible (*S*) become Exposed (*E*) after their interaction with the Infected population. The virus is in the incubation period within the Exposed population, until the onset of symptoms. The Exposed population show no symptoms of the virus, although they can transmit the virus to the Susceptible population. The time period that a person remains in the Exposed compartment follows the Erlang distribution with parameters k = 5 and λ = 5/5.2 = 0.96. According to this distribution the mean is 5.2 days, the 95% confidence interval is between 1.24 days and 9.88 days, and the 99% confidence interval is between 0.71 days and 12.6 days, which is aligned with the estimated incubation period reported in the literature [9]. The transmission rate of the Exposed population is affected by public health measures.
- After the incubation period, a person should be classified as either an asymptomatic, mild case, or a moderate/severe case. We assumed that 30% of the infected population would be asymptomatic, 55% mild cases, and 15% moderate/severe cases according to Lauer et al. [9]. There are three distinct behaviors connected to this classification:
  - Asymptomatic carriers (*A*) are mainly undetected and able to transmit the virus at the normal transmission rate (*β*). The only way to detect the asymptomatic carriers is through testing. Also, the transmission rate of asymptomatic individuals is affected by measures such as physical distancing.
  - Mild cases are isolated at home or in designated isolation centers (*I*) for 14 days on average. Mild cases may be infectious, but with a lower transmission rate than normal (*β_I_*) due to isolation and their transmission rate is affected by the government’s intervention measures.
  - Moderate and severe cases are admitted to hospitals (*H*) for 18 days on average. Depending on severity, they are assigned to regular beds or intensive care unit beds. We assumed the hospitalized percentage as 5.5 × CFR (case fatality ratio) while we assumed that the hospitalized population is not able to transmit the virus due to stringent isolation.

Using a similar approach to the SEIHR model [12], our model considers that the hospitalized patients have different behaviors from the rest of the infected patients.

- The Removed compartment (*R*) includes
  - Deaths: a percentage given by the CFR and the population age structure
  - Recovered patients: The remaining cases, who have cleared the infection and developed antibodies against the virus. In the context of our modelling, we consider the recovered patients immune to the virus thereafter.

### 2.1. Government Intervention: Public Health Measures

The government in every country has two main tools to manage the pandemic. One is to issue laws related to public health measures (such as mask wearing, physical distancing, and isolation), and the other is testing. The government may issue laws to mandate mask wearing in public places and increase the practice of physical distancing, and therefore, reduce the transmission rate. In our case we have taken a number of government actions and hypothetically assumed their impact from 0 (no social interaction) to 1 (full social interaction) as shown in Table 1. A similar approach was recently used to quantify the effect of physical distancing in another study [13].

### 2.2. Government Intervention: Testing

The other important part of the government intervention is testing. Tested positive people are found in the Exposed and Asymptomatic compartments. The testable population is considered the whole population except the people who have been diagnosed positive any time in the past. We assume that once they are found positive, they are immediately sent to designated isolation centres (or specific cases sent to home isolation) or the severe cases are hospitalized, where their transmission rate drops significantly. The tested positive cases increase the total cases for the day they are found positive. Table 2 shows the structure of the testing assumption employed by our model.

### 2.3. Model Definition

**Figure 1:**
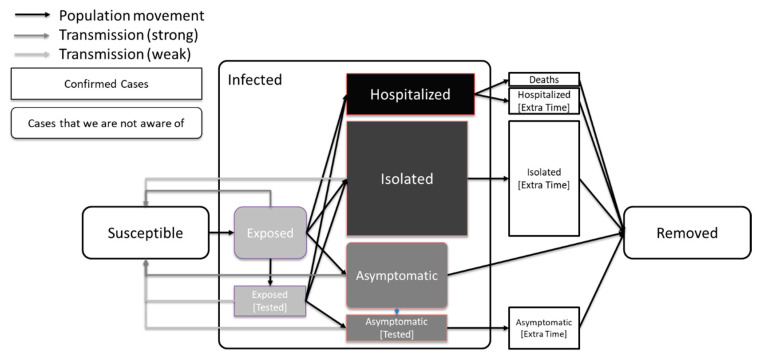
Model definition. The most infectious compartments are those of the Exposed and Asymptomatic carriers. Less infectious compartments are those of Isolated and tested people due to subsequent isolation. In this graph we also show that the average time of a person in the Exposed compartment is less than the average time of a person in the Asymptomatic compartment, which is in turn less than the average time of a person in the Isolated or Hospitalized compartments [9].

A schematic diagram of our model is shown in Figure 1. Table 3 shows the parameters used for the SEAHIR model.

The equations that define our model are below and Table 3 provides the description and value of the parameters.

$$\begin{array}{cc} \dot{S}= & -S\left(\min\left(\beta_{I},\beta_{t}\right)\left(I+E_{T}+A_{T}\right)+\beta_{t}\left(E+A\right)\right)/N\\ {\dot{E}}_{1}= & S\left(\min\left(\beta_{I},\beta_{t}\right)\left(I+E_{T}+A_{T}\right)+\beta_{t}\left(E+A\right)\right)/N-E_{1}/\alpha_{I}-P_{E\left[1\right]}\left(t\right)\\ {\dot{E}}_{i}= & E_{i-1}/\alpha_{I}-E_{i}/\alpha_{I}-P_{E\left[i\right]}\left(t\right),\;i=2\ldots I\\ {\dot{E}}_{T,1}= & P_{E\left[1\right]}\left(t\right)-E_{T,1}/\alpha_{I}\\ {\dot{E}}_{T,i}= & P_{E\left[i\right]}\left(t\right)+\;E_{T,i-1}/\alpha_{I}-E_{T,i}/\alpha_{I},\;i=2\ldots I\\ {\dot{A}}_{1}= & pE_{I}/\alpha_{I}-A_{1}/\zeta_{J}-P_{A\left[1\right]}\left(t\right)\\ {\dot{A}}_{j}= & A_{j-1}/\zeta_{J}-A_{j}/\zeta_{J}-P_{A\left[j\right]}\left(t\right),\;j=2\ldots J\\ {\dot{A}}_{T,1}= & P_{A\left[1\right]}\left(t\right)+pE_{T,I}/\alpha_{I}-A_{T,1}/\zeta_{J}\\ {\dot{A}}_{T,j}= & P_{A\left[j\right]}\left(t\right)+A_{T,j-1}/\zeta_{J}-A_{T,j}/\zeta_{J},\;j=2\ldots J\\ {\dot{A}}_{ET}= & A_{T,1}/\zeta_{J}-A_{ET}/\delta \\ {\dot{I}}_{1}= & \left(1-p\right)\left(1-s\right)\left(E_{I}+E_{T,I}\right)/\alpha_{I}-I_{1}/\gamma_{K}\\ {\dot{I}}_{k}= & I_{k-1}/\gamma_{K}-I_{k}/\gamma_{K},\;k=2\ldots K\\ {\dot{I}}_{ET}= & I_{K}/\gamma_{K}-I_{ET}/\delta \\ {\dot{H}}_{1}= & \left(1-p\right)s\left(E_{I}+E_{T,I}\right)/\alpha_{I}-H_{1}/\gamma_{L}\\ {\dot{H}}_{l}= & H_{l-1}/\gamma_{L}-H_{l}/\gamma_{L},\;l=2\ldots L\\ {\dot{H}}_{ET}= & H_{L}/\gamma_{L}-H_{ET}/\delta \\ \dot{R}= & A_{J}/\zeta_{J}+A_{ET}/\delta +I_{ET}/\delta +H_{ET}/\delta \end{array}$$

where $S+E+E_{T}+A+A_{T}+A_{ET}+I+I_{ET}+H+H_{ET}+R=N$, $\dot{S}+\dot{E}+{\dot{E}}_{T}+\dot{A}+{\dot{A}}_{T}+{\dot{A}}_{ET}+\dot{I}+{\dot{I}}_{ET}+\dot{H}+{\dot{H}}_{ET}+\dot{R}=0$, $\beta_{t}=\beta G\left(t\right)$, $\alpha ,\gamma_{I},\gamma_{H},\zeta ,\delta >0$, 0<p,s<1, $E=\sum E_{i}$, $A=\sum A_{j}$, $I=\sum I_{k}$, $H=\sum H_{I}$, $\alpha_{I}=\frac{\alpha }{I}$, $\gamma_{K}=\frac{\gamma_{I}}{K}$, $\gamma_{L}=\frac{\gamma_{H}}{L}$, $\zeta_{J}=\frac{\zeta }{J}$. We have set I=J=K=L=5. The subscript T denotes the tested positive cases, and the subscript ET denotes the extra time. Regarding the tested positive people, we assume that their compartmental distribution follows the compartmental distribution of the existing carriers at day t. Hence, the following relations apply (dropping the (t) notation from E, A, E_i_, and A_j_ for simplicity):

$$\begin{array}{l} P\left(t\right)=P_{E}\left(t\right)+P_{A}\left(t\right)\\ P_{E}\left(t\right)=P\left(t\right)\frac{E}{E+A}=\sum P_{E\left[i\right]}\left(t\right)\\ P_{A}\left(t\right)=P\left(t\right)\frac{A}{E+A}=\sum P_{A\left[j\right]}\left(t\right)\\ P_{E\left[i\right]}\left(t\right)=P_{E}\left(t\right)\frac{E_{i}}{E}=\;P\left(t\right)\frac{E_{i}}{E+A}\\ P_{A\left[j\right]}\left(t\right)=P_{A}\left(t\right)\frac{A_{j}}{A}=P\left(t\right)\frac{A_{j}}{E+A} \end{array}$$

### 2.4. Possible Challenges Caused by the Asymptomatic Patients

A significant part of the complexity of this model is due to the expected presence of the Asymptomatic patients who become infected but not tested. The official statistics of confirmed cases will only report the people who have tested positive i.e., the number of confirmed cases and not infections. We assume that Isolated and Hospitalized patients are tested positive by default. Therefore, we need to make a strict distinction between the confirmed infected cases that are reported in the official statistics, and the infected cases as a superset that includes both the confirmed infected cases and asymptomatic not tested cases. Following the above issue, the Recovered compartment may contain people who have been asymptomatic but not tested in the past. These people might not be counted in the official statistics as due to a lack of symptoms they might not have been tested. The official statistics show only the confirmed recovered (*R*_C_) cases, which only consists of previously diagnosed laboratory-confirmed cases who have now recovered.

## 3. Results

With all the required equations in place, we can proceed with the estimation of the free parameters. These free parameters are the base transmission rate (*β*), the transmission rate in Isolation (*β_I_*), and the average extra time people spent in isolation or in the hospital (*δ*) without being infectious.

The publicly available data for UAE cases are announced on a daily basis. They consist of the total confirmed cases (*C*), the total recoveries (*R*), and the total deaths (*D*). There is no further breakdown on the total confirmed cases as to whether they are hospitalized or isolated. Therefore, we will consider the actual confirmed cases (*C*)-(*R*)-(*D*). The actual confirmed cases will be optimized against the sum of our estimations based on our model to isolated (*I*), isolated in extra time (*I_ET_*), hospitalized (*H*), hospitalized in extra time (*H_ET_*), exposed tested (*E_T_*), and asymptomatic tested (*A_T_*). A practical consideration running this part of the optimization is to be more lenient to the negative error. We define the error as (actual)—(estimation), therefore, negative error means (actual) < (estimation). By considering the estimations to be greater than the actual data, we assume that there are undetected patients. This is expected in any population due to the nature of the virus and the possibility of having asymptomatic not tested patients.

Another layer of calculations focuses on the recoveries. While in our equations we calculated all the recoveries, the publicly available data are the recoveries of the confirmed cases. This means that we need to estimate the recoveries of the confirmed cases only. We can achieve so by considering the following equation:

$${\dot{R}}_{A}=A_{T,J}/\zeta_{J}+I_{ET}/\delta +H_{ET}/\delta$$

where the subscript A stands for Aggregated. Then the confirmed recovered should be $R_{C}=\left(1-m\right)R_{A}$, and the deaths should be $D=mR_{A}$, with m being the mortality rate given above.

**Figure 3:**
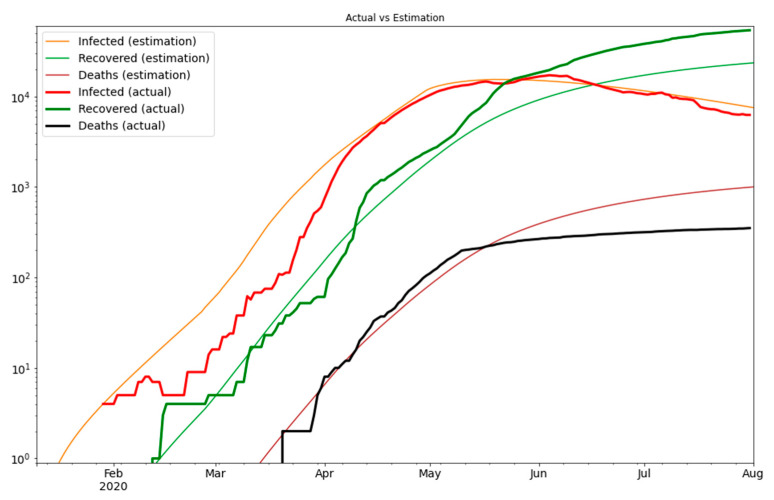
The optimization result on the logarithmic scale.

**Figure 4:**
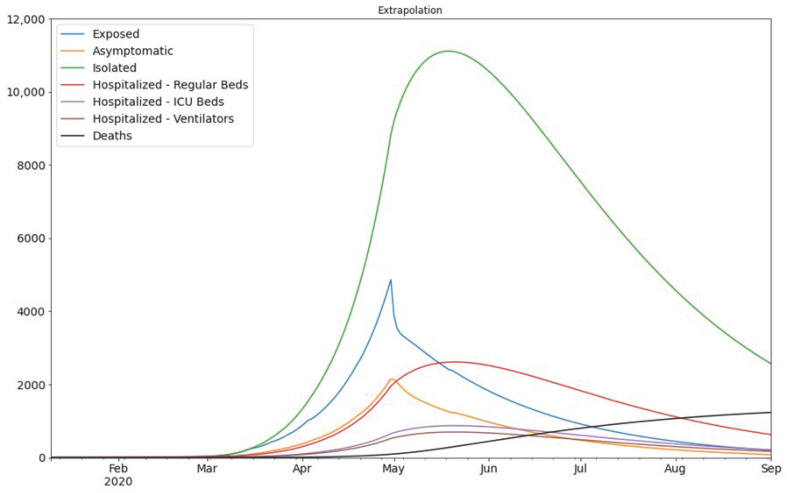
The extrapolation of the result of Figure 3. The assumed division of infected population is as follows: 30% of the Infected population would be asymptomatic, 55% mild cases, and 15% hospitalized (which are further divided into three sub-categories) according to Lauer et al. [9].

Finally, we need to consider the logarithmic scale as the scale of the error measurement. This is because the growth of the new cases is exponential over time. Figure 3 shows that our estimate closely matches the actual values, therefore, it gives us a strong indication that our model is a reliable tool for predictions. Based on our model we can predict the number of exposed and asymptomatic people in the UAE, and we can forecast the number of future cases more accurately. In Figure 4 we see the extrapolated results. 

## 4. Discussion

We have presented an epidemiological compartmental model inspired by the well-known SEIR and modified according to the unique features of the COVID-19 outbreak. This model represents a useful tool which can be used for guiding public health policies and making projections for healthcare needs during the ongoing COVID-19 pandemic. It also permits decision-makers and researchers to accurately predict the transmission rate and evaluate the effectiveness of government interventions, and it provides an accurate estimate of the Exposed and Asymptomatic carriers. The SEAHIR model was used by decision-makers in Dubai’s COVID-19 Command and Control Center to make timely decisions on developing testing strategies, increasing healthcare capacity, and implementing interventions to contain the spread of the virus. However, with modification to the parameters we believe that it can also be applied to other countries. With the opening of countries from strict lockdown measures and implementation of relaxation of restrictions, a second wave of infections is currently being experienced in several countries. Hence, the need for accurate prediction models, such as the one described in this paper, to help mitigate against the occurrence of new waves of infection as well to enable proper planning for the disbursement of healthcare resources.

## 5. Future Work

Through the development and optimization of the SEAHIR model, we have identified several areas for future work in SARS-CoV-2 research, including:

- Estimating the public reaction on physical distancing. The more deaths announced, the more we expect people to take measures for personal hygiene and distancing. That said, population compliance with infection control and public health measures might reduce over time due to pandemic fatigue.
- Modelling the mortality and transmission rate of the virus, as it mutates over time, and embed the findings into the SEAHIR model. Over time we expect that the more prevalent viral strain in the population may be more transmissible but less pathogenic and lethal (virulent).
- Optimizing the parameters of government intervention on physical distancing. To date, the physical distancing modifiers imposed by the government were chosen arbitrarily. A focused study would be useful on how these measures can be quantified based on the available data.
- The viral load is not modelled in this work. Given that we have adequate evidence that in the future, we may assume that the viral load in a patient gradually increases during the incubation period, affecting the test results and the transmission rate. Our choice of Erlang distribution can easily model the viral load by assigning a gradually increasing percentage in each of the *E_i_* phases the patient belongs to.
- The current version of our model does not account for re-infections. The available scientific literature on the duration of immunity following SARS-CoV-2 infection, risk of re-infection, and severity of disease following re-infection is sparse. Evidence on the duration of immunity will emerge and become more robust over the next 6–12 months and future models (or adaptations of the SEAHIR model) may be able to include parameters related to re-infection.
- Numerous countries, including the UAE, have initiated population-wide vaccination campaigns and future models may be able to quantify the effect of different vaccines on transmission dynamics when sufficient data are available.

## 6. Conclusions

In conclusion, the novel six-compartment SEAHIR model builds upon earlier SEIR infectious disease models and incorporates the unique dynamics of the virus by further dividing the Infected compartment into Asymptomatic, Isolated, and Hospitalized compartments. The SEAHIR model was used to effectively manage the COVID-19 pandemic in Dubai (UAE) and could be utilized by decision-makers and researchers in other countries for current or future pandemics with similar viral transmission dynamics.

## Figures and Tables

**Figure 2:**
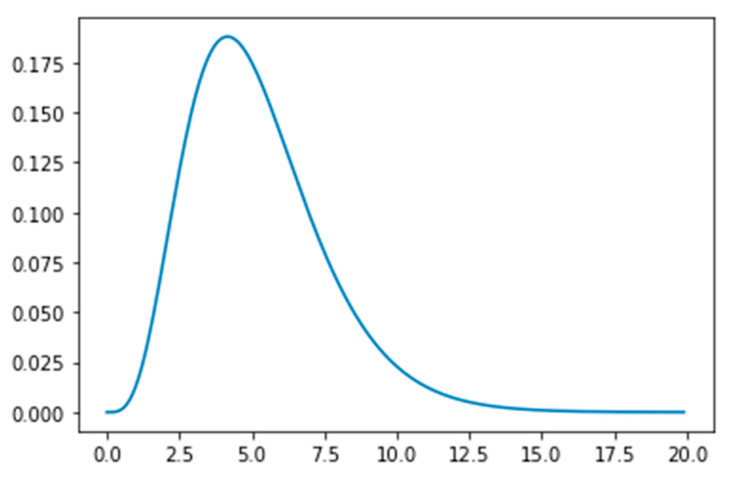
The Erlang distribution with parameters k = 5 and λ = 5/5.2 = 0.96, showing the time (in days) an exposed person spends in incubation period.

**Table 1:** The UAE Government public health intervention on mask wearing and physical distancing. By enforcing mask wearing in public places and physical distancing, the UAE Government introduces public health interventions and we quantify the effects of these measures as multiplier *G(t)*, which finally reduces the base transmission rate (*β_t_*). The values are assumed, and *G(t)* decreases as more measures are taken.

Date	*G(t)*	Description
2020-03-01	0.95	Nurseries closed ^1^
2020-03-08	0.90	Schools and universities closed ^2^
2020-03-15	0.85	Gyms, parks, cinemas, and pools closed ^3^
2020-03-16	0.80	Mosques and other worship places closed ^4^
2020-03-17	0.75	Suspension of issuing entry visas ^5^
2020-03-23	0.70	Malls, shopping centers, restaurants closed ^6^ Passenger flights suspended ^7^
2020-04-04	0.65	Restrictions on non-essential movement (extension of the disinfection program to 24 h a day in Dubai) ^8^
2020-05-01	0.40	Physical distancing measures

^1^https://www.khaleejtimes.com/coronavirus-outbreak/live-number-of-coronavirus-patients-in-uae-increases-to-21-1 (accessed date: 2 March 2021); ^2^ https://www.khaleejtimes.com/coronavirus-outbreak/uae-schools-to-close-for-four-weeks-as-precaution-against-covid-19-coronavirus (accessed date: 2 March 2021); ^3^ https://www.khaleejtimes.com/coronavirus-outbreak/coronavirus-in-uae-cinemas-in-dubai-temporarily-closed- (accessed date: 2 March 2021); ^4^ https://www.khaleejtimes.com/coronavirus-outbreak/coronavirus-prayers-at-uae-mosques-other-places-of-worship-suspended-for-four-weeks- (accessed date: 2 March 2021); ^5^ https://www.khaleejtimes.com/coronavirus-outbreak/coronavirus-uae-visa-suspension-begins-today (accessed date: 2 March 2021); ^6^ https://www.khaleejtimes.com/coronavirus-outbreak/coronavirus-uae-to-close-shopping-malls-for-two-weeks (accessed date: 2 March 2020); ^7^ https://www.khaleejtimes.com/coronavirus-outbreak/covid-19-uae-suspends-all-passenger-and-transit-flights (accessed date: 2 March 2020); ^8^ https://gulfnews.com/uae/government/covid-19-disinfection-drive-extended-to-24-h-in-dubai-1.1586019164814 (accessed date: 3 March 2020).

**Table 2:** The assumed positive cases detected over time that can result from random tests. The numbers below are only assumptions, since we do not have the official test data about positive cases found on people randomly sampled from the population.

Date	Positive
2020-02-27	1
2020-03-03	2
2020-03-09	3
2020-03-10	5
2020-03-29	20
2020-04-20	60
2020-05-01	80
2020-05-10	90
2020-05-20	40

**Table 3:** The parameters used for the SEAHIR model.

Name	Notation	Value	Description
Initial population size	*N*	9,366,829	Given by the UAE Federal Competitiveness and Statistics Authority [14]
Transmission rate	*β*	0.1466 ± 0.0004 *	Estimated in optimization based on publicly available data, see Section 3 below. This is the estimated base transmission rate for the UAE
Transmission rate in isolation	*β_I_*	0.0623 ± 0.0011 *	Estimated in optimization based on publicly available data, see Section 3 below. This parameter is modelling the effects of (1) the nosocomial infections in the isolation facilities, (2) expected infected healthcare workers who might then move/live in the community and contribute to community-transmission, (3) infecting housemates, and (4) possible breaking the isolation rules
Base transmission rate multiplier	*G(t)*	See Table 1 (physical distancing). The values of *G(t)* vary over time due to the government measures implemented. Before 1 March 2020 we set *G(t)* = 1. From 1 March 2020 and up to 4 March 2020, we set *G(t)* = 0.95. From 5 March 2020 and up to 14 March 2020, we set *G(t)* = 0.90, and so on. From 1 March 2020 onwards, we set *G(t)* = 0.40.	
Positive tested cases	*P(t)*	See Table 2 (testing). The values of *P(t)* vary over time. For dates before 27 February 2020 we assume no positive people would be found if a member of the population was randomly selected and tested, so *P(t)* = 0. For the dates not shown we assume the number of the last date shown, up to the specific date we are interested in. For example, for the 25 March 2020 we consider *P(t)* = 5 positive people found, because the last date up to 25 March 2020 shown is 10 March 2020 that indicates 5 positive people found based on our assumption in Table 2.	
Mean incubation period (days)	*α*	5.2	Based on data from [9,15,16]
Average period the asymptomatic carriers are considered infectious (days)	*ζ*	9.0	Assumed based on data from [9]
Average period in isolation (days)	*γ_I_*	14.0	Assumed based on data from [9,16]
Average period in hospital (days)	*γ_H_*	18.0	Assumed based on data from [16]
Average extra time in hospital or isolation (days)	*δ*	31.1 ± 8.0 *	Estimated in optimization based on publicly available data, see below. Some people stay in hospital or isolation for additional time, until they are finally discharged. During this period, they are not infectious anymore and are only recovering
Mortality rate	*m*	2.4%	Given from the population age structure and the CFR table [15]
Hospitalization need	*h*	5.5	Assumed based on data from [16]
Percentage of the Exposed population requiring hospitalization	*s*	13.2%	*m·h*
Asymptomatic percentage	*p*	30%	Assumed

Additional notes: • We assume that the first exposed person (or group of people) arrived in the UAE on 16 January 2020 according to published viral genomics research [17,18,19]. • Up to 27 February 2020 all patients were assumed to be hospitalized. • The Exposed, Asymptomatic, Isolated and Hospitalized population is assumed to follow the Erlang (k, λ) distribution in these compartments based on the reported cases in the UAE. The Erlang distribution is supported by recent literature [20]. Note that the Erlang distribution is a special case of the Gamma (k, λ) distribution, with k being an integer. What makes the Erlang distribution a better choice compared to the Gamma distribution is the fact that a random sample from the Erlang distribution can be expressed as the sum of k random samples of an exponential distribution with parameter λ. The connection and the stability improvement of the SEIR model using the Erlang distribution is shown in [20]. In all cases we assumed k = 5. Figure 2 shows the Erlang distribution for the incubation period. * denotes the upper and lower 95% limits.

## Data Availability

All the data used are publicly available and the source cited in the manuscript. Please contact the corresponding authors for more details.
